# Assessing Work Functioning in Patients with Persistent Low Back Pain: Exploring the Structural Validity of the Work Rehabilitation Questionnaire

**DOI:** 10.1007/s10926-023-10157-9

**Published:** 2023-12-15

**Authors:** Anders Hansen, Ole Steen Mortensen, Reuben Escorpizo, Karen Søgaard, Jens Søndergaard, Berit Schiøttz-Christensen, Henrik Hein Lauridsen

**Affiliations:** 1 Medical Research, Spine Centre of Southern Denmark, Lillebaelt Hospital, University Hospital of Southern Denmark, Østre Houghvej 55, 5500 Middelfart, Denmark; 2 Department of Regional Health Research, University of Southern Denmark, Odense, Denmark; 3 Department of Occupational and Social Medicine, Holbæk Hospital, Part of Copenhagen University Hospital, Holbæk, Denmark; 4 Department of Public Health, Section of Social Medicine, University of Copenhagen, Copenhagen, Denmark; 5 Department of Rehabilitation and Movement Science, The University of Vermont, Burlington, VT USA; 6 Swiss Paraplegic Research, Nottwil, Switzerland; 7 Department of Sports Science and Clinical Biomechanics, University of Southern Denmark, Odense, Denmark; 8 Research Unit for General Practice, Department of Public Health, University of Southern Denmark, Odense, Denmark

**Keywords:** Occupational rehabilitation, Low back pain, Psychometric, Exploratory factor analysis

## Abstract

**Purpose:**

Assessing work functioning in patients with persistent low back pain (LBP) is important for understanding their ability to engage in work-related activities. This study aims to evaluate the item characteristics, factor structure, and internal consistency of the Work Rehabilitation Questionnaire (WORQ) in patients with persistent LBP.

**Methods:**

Four hundred and twenty-five individuals with LBP completed the WORQ. Item characteristics, exploratory factor analysis (EFA), and consistency were performed to identify the underlying factors.

**Results:**

Missing responses were < 2% for each item. The analysis revealed three factors: psychological wellbeing, physical functioning, and cognitive ability. The factors demonstrated strong internal consistency, with Cronbach’s alpha values ranging from 0.88 to 0.93 and McDonald’s Omega from 0.92 to 0.96. Fifteen items did not fit into any identified factors, suggesting their potential value in screening functioning levels beyond the factors.

**Conclusions:**

The WORQ is a valid instrument for evaluating work limitations in individuals with persistent LBP. Further research should assess its responsiveness to changes from interventions that target workability. Advancing this knowledge has the potential to promote work rehabilitation and improve the quality of life for patients with persistent LBP.

## Introduction

Low back pain (LBP) is a common condition that affects individuals across all age groups and occupations, particularly those with physical work demands [1]. Persistent LBP can have significant consequences, such as work absenteeism, early retirement, and negatively impacting the quality of life [2, 3]. To address work disability, work rehabilitation is an evidence-based approach to enhancing work participation for individuals with health-related impairments and disabilities [4]. In this context, accurate assessment of functioning levels is important, and various measures, including patient-reported outcomes [5], performance-based tests, and work simulations, are available [6] depending on the evaluation’s purpose and target population.

Questionnaires are the prevailing means of assessing function in work rehabilitation. Whilst single-item questions like the Work Ability Index single item [7] are commonly used for convenience, none of these fully captures the intricate interplay of multiple factors that influence an individual’s work functioning [8]. In contrast, multi-item questionnaires may enable a more comprehensive assessment, providing a detailed understanding of the factors affecting workability. Both generic and disease-specific questionnaires serve this purpose. Generic questionnaires typically evaluate impairments, limitations in daily tasks, and restrictions in participating in life events, offering a comprehensive understanding of the overall health state. However, they may lack specificity in capturing how a health condition explicitly affects the level of functioning [9]. On the other hand, disease-specific questionnaires focus on a particular medical condition and provide detailed insights into how the condition impacts the domain under investigation. They may, however, not accurately reflect the overall health state of individuals affected by multiple conditions and may lack generalizability to understand the general health state.

The Work Rehabilitation Questionnaire (WORQ) [10] is a generic instrument based on the International Classification of Functioning, Disability, and Health (ICF) [11] framework by the WHO to provide a comprehensive understanding of an individual’s work functioning. The ICF is a comprehensive framework that considers various dimensions of health beyond just health conditions. It considers physical, psychological, mental, social, and contextual factors to provide a holistic understanding of an individual’s health and lived experience. Whilst previous research has established the WORQ as a valuable measurement tool across diverse patient populations [12–17], exploratory factor analyses (EFA) have identified an alternative factorial structure compared to the original [13]. This finding underscores the necessity for a meticulous psychometric evaluation tailored to specific patient demographics. Through examining individual item characteristics, exploring factor structures, and assessing reliability in individuals with persistent LBP, this study contributes to the ongoing refinement of WORQ.

## Methods

The study was performed at the Spine Centre of Southern Denmark, an outpatient hospital department evaluating spine-related conditions.

The study population consisted of patients with persistent LBP. The inclusion criteria were ages between 18 and 65. Exclusion criteria were early retirement, not being available for the labour market, or a score of 0 on a 0–10 scale for the question, “How likely is it that you will be working in six months?” (0 meaning “completely unlikely”), as these patients were considered outside of our target population, i.e., had some likelihood of returning to work. The determination of eligibility was carried out by executing a rule-based algorithm using self-reported data from the local clinical patient registry known as “My Spine Data” (MiRD), an extension of SpineData [18].

### Procedure

Written informed consent was obtained after the clinical evaluation at the Spine Centre, and the study participants completed the WORQ electronically on-site or through the MiRD registry. At least 400 participants were included to obtain a participant-to-WORQ-item ratio of 10:1, ensuring the stability of the variance–covariance matrix in the factor analyses [19].

### Variables

Demographic and clinical information included sex, age, body mass index, LBP intensity, disability, and overall health state registered in the MiRD system. The WORQ consists of 40 items evaluating functioning levels, including 18 on body functions and 22 on activity and participation. The items are scored on an 11-point numeric rating scale (0–10), with a higher score indicating a more significant problem or difficulty. A total score of 40 items (0–400) can be calculated, reflecting the individual’s work ability [10]. Additionally, the WORQ includes two questions assessing the time needed for self-care and therapy. These items were excluded from the validation process because of their narrative character and lack of properties allowing inclusion in the factor analysis. See www.myworq.org for the self-reported full version of the WORQ used.

### Statistics

Demographic and clinical information was presented using descriptive statistics. The distribution of responses for each WORQ item was summarised, including details on the percentage of missing item scores, skewness, and kurtosis. Missing responses below 3% for an item was considered acceptable [20].

To determine WORQ’s factor structure, an EFA was performed. A correlation matrix was constructed using Pearson’s correlation coefficient and complete cases. Based on this matrix, the determinant, Bartlett’s test of sphericity, and the Kaiser–Meyer–Olkin (KMO) measure were assessed to evaluate the suitability of the data for EFA. The determinant provides information on the multicollinearity of variables; Bartlett’s test assessed the appropriateness of the correlation matrix for factor analysis; and the KMO measure determined the sampling adequacy [21]. A Barlett’s test with a significant *p* value of < 0.05 and a KMO of at least 0.5 is necessary to proceed with EFA.

A principal axis factor analysis was conducted for the EFA [22], and the number of factors was determined using Kaiser’s criteria by comparing eigenvalues from actual and randomly generated data [23]. Cattell’s method plots the eigenvalues in decreasing order and retains as many factors as possible as long as there are eigenvalues above the elbow of the plot [24]. Horn’s parallel analysis modifies Cattell’s scree plot by comparing the observed eigenvalues to eigenvalues from random data, retaining factors with eigenvalues that exceed the simulated eigenvalues [25].

The factor analysis involved an iterative process. Oblique rotation with a common factor method was used, allowing the factors to be correlated [26]. The initial step involved excluding items with factor loadings below 0.3 and communalities below 0.25. This lower threshold was implemented to maintain inclusivity whilst refining the factor structure. Subsequent analyses were conducted to eliminate items with inadequate factor loading (< 0.5) and communalities (< 0.4), thereby [27]. If any item was removed, each phase of the EFA process was re-iterated [28]. To ensure theoretical congruence, items that aligned conceptually with the construct of interest were retained, even if their factor loading fell below the 0.5 threshold. Cross-loading items were carefully placed into the factor structure by clinical reasoning and alignment with the underlying ICF codes. Internal consistency was assessed using Cronbach’s alpha and McDonald’s Omega coefficients. Cronbach’s alpha examined the interrelatedness of items within each factor, with values exceeding 0.7 indicating reliable measurement [29]. McDonald’s Omega, a more recent and robust measure of internal consistency, considers the proportion of true score variance relative to the total observed score variance [30]. This dual approach comprehensively evaluates the measurement’s reliability. Outliers were identified in individuals with complete information using the interquartile range (IQR) method. Potential outliers were defined by sum scores in each domain lying beyond the boundaries of Q1 − 1.5 × IQR and Q3 + 1.5 × IQR. A sensitivity analysis excluding these individuals was conducted if outliers were identified. Analyses were made in R version 4.3 [31] using the psych [32] packages.

## Results

Between December 2021 and 2022, 532 patients consented to the trial. Of these, 425 participants completed the WORQ. Besides a negligible gender difference, there were no statistically significant differences in demographic or self-report data between participants who completed the WORQ and those who did not (Table 1). No items met the 3% threshold, indicating no violation of the assumption of missingness (Table 2). The determinant of the correlation matrix was 7.45, indicating no multicollinearity issues amongst the variables. Bartlett’s test of sphericity gave a *p* value of < 0.00, and the KMO value was 0.93, indicating a high level of factor adequacy. Meeting all the prerequisites, the test statistics met the necessary criteria for factor analysis.

**Table 1:** Demographic and clinical characteristics differences between individuals completing WORQ and those who did not

Characteristic	Completed WORQ, *N* = 425^a^	Not completed WORQ, *N* = 107	Differences^b^	96% CI^b,c^	*p* value^b^
Age	53 (43, 59)	50 (39, 58)	2.4	− 0.37, 5.2	0.09
Sex (female)	269 (63%)	52 (52%)			0.05
BMI (kg/m^2^)	27.6 (24.3, 31.7)	27.4 (24.1, 31.6)	0.4	− 0.86, 1,7	0.5
LBP intensity (0–10)	5 (4,7)	5 (3.7)	0.2	− 0.34, 0.72	0.5
Oswestry disability index (0–100)	30 (20, 40)	28 (18, 38)	1.2	− 2.2, 4.6	0.5
EQ-5D VAS (0–100)	57 (39, 74)	59 (40,70)	1.8	− 4.2, 4.6	> 0.9

^a^Median (IQR); *n* (%)

^b^Welch Two Sample t-test: Standardized Mean Difference: Two Sample test for equality of proportions or Persons *χ*^2^

^*c*^*CI* Confidence Interval

**Table 2:** Distribution of the Work Rehabilitation Questionnaire (WORQ) scores at baseline based on 524 patients with low back pain

Item	Overall, in the past week, to what extent did you have problems with…	0	1	2	3	4	5	6	7	8	9	10	% missing	Skewness	Kurtosis
1	… not feeling rested and refreshed during the day?	20	15	32	33	25	55	64	78	60	22	21	0.0	− 0.45	− 0.57
2	… sleeping, such as falling asleep, waking up frequently during the night or waking up too early in the morning?	25	18	37	25	17	37	51	52	75	51	36	0.2	− 0.52	− 0.85
3	… remembering to do important things	97	47	59	25	28	58	31	35	31	6	7	0.2	0.38	− 1.07
4	… your usual daily activities because you felt sad or depressed?	155	61	44	32	23	30	24	21	13	9	9	0.9	0.97	− 0.18
5	… your usual daily activities because you felt worried or anxious?	141	62	54	35	27	30	26	16	19	5	8	0.5	0.94	− 0.17
6	… being irritable?	59	51	49	46	48	51	42	40	21	13	4	0.2	0.24	− 0.96
7	… your temper?	92	69	51	49	31	36	37	30	17	9	4	0.0	0.58	− 0.78
8	… your self− confidence?	109	55	47	33	35	41	34	31	19	14	7	0.0	0.53	− 0.90
9	… thinking clearly?	91	76	53	33	38	38	44	25	16	7	3	0.2	0.55	− 0.83
10	… analysing and finding solutions to problems in day-to-day life?	105	73	50	46	32	39	31	27	12	5	5	0.0	0.69	− 0.53
11	… hearing?	229	52	38	29	15	27	13	12	6	2	1	0.2	− 0.81	0.13
12	… keeping your balance whilst maintaining a position or during movement?	81	61	58	41	36	40	24	37	31	6	6	0.9	0.49	− 0.93
13	… bodily aches or pains?	2	6	9	19	21	37	31	76	96	51	77	0.0	− 0.81	0.13
14	… general endurance when performing physical activities?	17	28	30	26	24	61	40	58	71	33	35	0.5	− 0.39	− 0.85
15	… muscle strength?	47	42	39	35	40	52	40	46	39	28	16	0.2	0.03	− 1.13
16	… skin problems, such as broken skin, ulcers, bedsores and thinning of skin?	250	56	28	21	9	19	14	7	8	5	4	0.9	1.85	2.59
17	… learning a new task (e.g., learning a new game, learning how to use the computer, learning how to use a tool, etc.)?	182	74	41	26	20	26	15	15	13	2	7	0.9	1.33	0.82
18	… focusing attention on a specific task or e.g. filtering out distractions such as noise?	127	75	49	39	27	29	25	29	12	7	5	0.2	0.86	− 0.36
19	… reading?	204	54	38	26	23	27	17	13	9	8	4	0.5	1.3	0.69
20	… making decisions?	168	76	45	24	22	31	23	14	9	8	3	0.5	1.16	0.30
21	… starting and completing a single task such as making your bed or cleaning up your desk or workplace?	168	37	44	49	24	29	23	22	16	9	4	0.5	0.88	− 0.35
22	… carrying out your daily routine or day to day activities?	81	38	62	51	31	50	29	32	20	16	12	0.7	0.47	− 0.81
23	… handling stress, crises, or conflict?	112	68	55	34	29	36	20	21	28	14	7	0.2	0.74	− 0.65
24	… understanding body gestures, symbols and drawings?	222	70	37	26	17	26	6	9	5	4	1	0.5	1.68	2.22
25	… starting and maintaining a conversation?	246	61	39	20	13	20	10	8	2	3	2	0.2	1.91	3.23
26	… using communication devices such as using a telephone, telecommunication devices, and computers?	258	73	34	17	8	10	2	5	4	2	4	1.9	2.7	7.75
27	… lifting and carrying objects weighing up to 5 kg?	97	48	44	29	27	39	21	35	39	28	16	0.5	0.34	− 1.28
28	… lifting and carrying objects weighing more than 5 kg?	51	22	23	38	18	26	28	41	57	43	76	0.5	− 0.37	− 1.25
29	… fine hand use such as handling objects, picking up, manipulating and releasing objects using the hand, fingers, and thumb?	215	55	35	21	13	17	14	18	18	8	7	0.9	1.36	0.56
30	… walking a short distance (less than 1 km)?	182	55	41	20	17	27	11	11	20	20	17	0.9	1.1	− 0.16
31	… walking a long distance (more than 1 km)?	83	34	31	32	20	35	24	40	32	27	65	0.5	0.06	− 1.45
32	… moving around including crawling, climbing, and running?	22	21	25	24	28	42	32	50	56	50	73	0.5	− 0.50	− 0.89
33	… using transportation as a passenger?	193	51	40	18	25	29	15	18	15	10	8	0.7	1.14	0.12
34	… driving a car or any form of transportation?	191	58	38	22	27	16	15	17	17	6	15	0.7	1.25	0.38
35	… getting dressed?	116	60	60	42	28	36	31	18	18	11	3	0.5	0.75	− 0.52
36	… looking after your health such as maintaining a balanced diet, getting enough physical activity and seeing your doctor as needed?	139	54	53	42	30	29	26	20	15	10	7	0.0	0.87	− 0.23
37	… your relationships with people?	212	55	51	18	18	17	14	13	14	8	4	0.2	1.48	1.12
38	… having sufficient money to cover your cost of living?	266	57	24	11	7	11	7	16	9	8	8	0.2	1.96	2.65
39	… seeing and recognizing an object at arm's length?	348	10	35	10	3	6	3	3	5	0	0	0.5	3.26	11.26
40	… seeing and recognizing a person you know across the road (distance of about 20 m or 66 feet)?	310	12	40	19	8	15	9	4	1	2	0	0.9	2.19	4.16

**Fig. 1:**
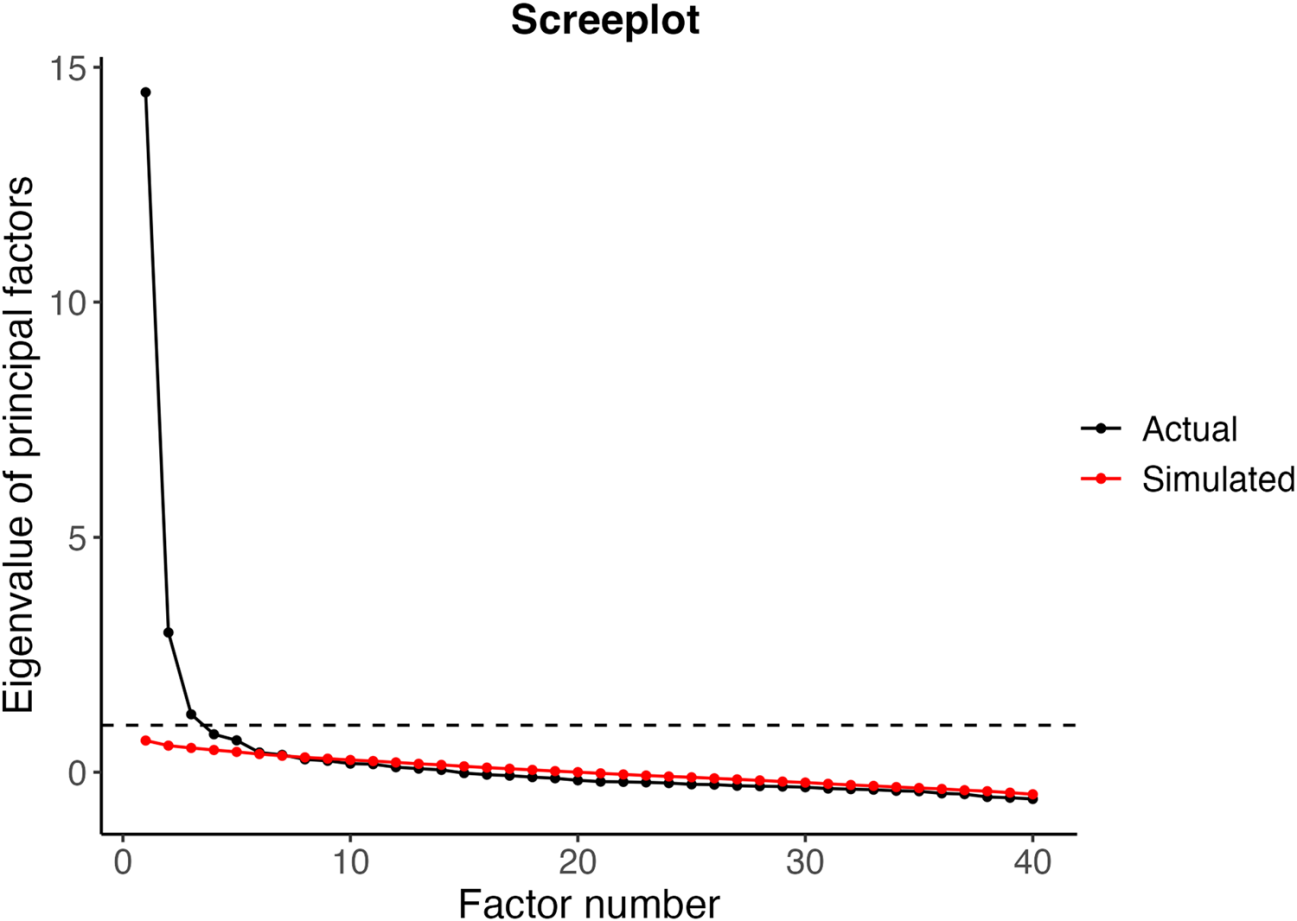
Scree plot displaying eigenvalues of principal factors. The x-axis represents the number of factors, whilst the y-axis represents the corresponding eigenvalues. The scree plot shows a steep decline in eigenvalues initially, indicating that a small number of factors explain most of the variance in the data. After a certain point, the eigenvalues level off, suggesting that additional factors contribute relatively less to the overall variance

**Fig. 2:**
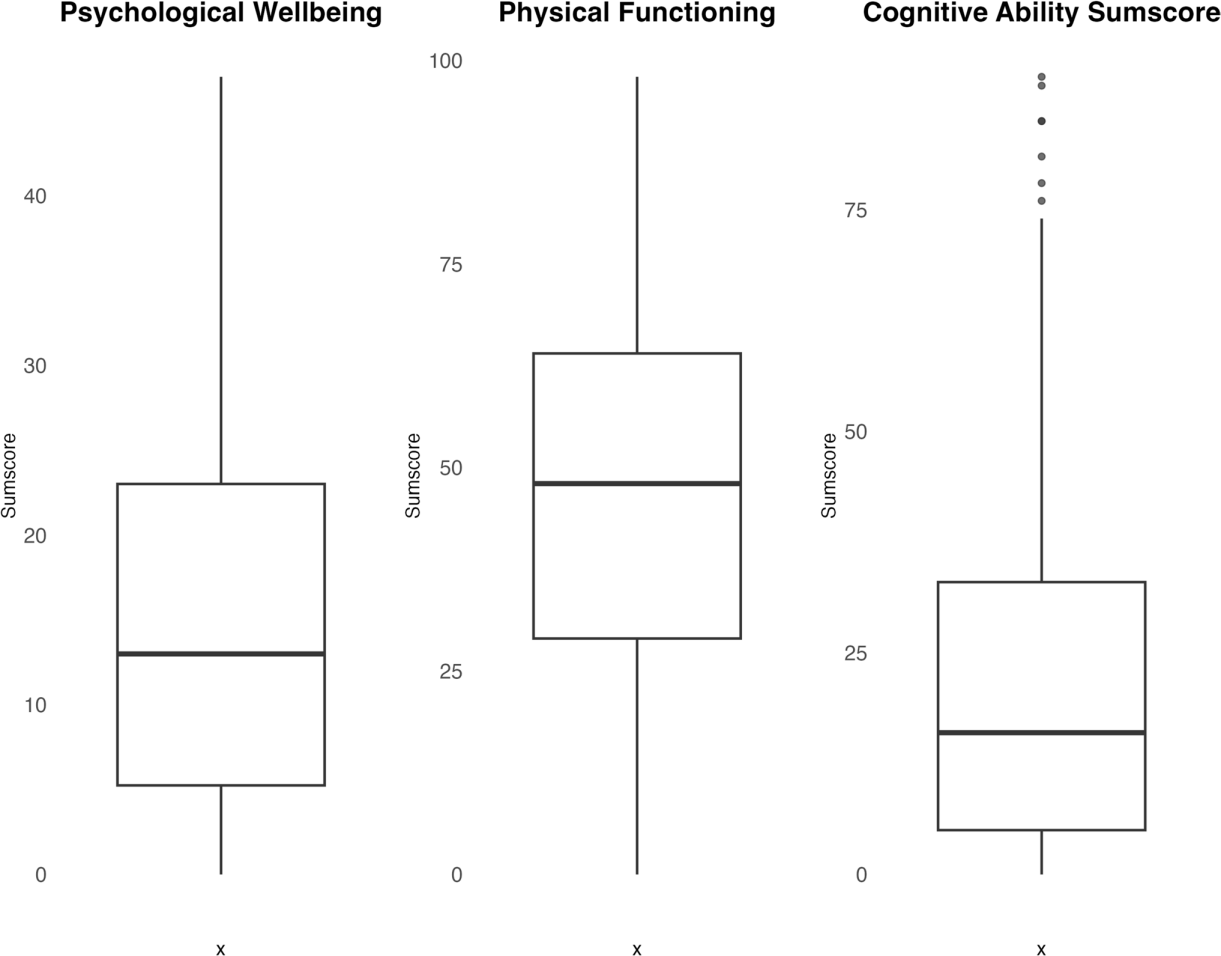
Boxplot depicting the distribution of sumscores for three domains: Psychological wellbeing, Physical functioning, and Cognitive ability. Each boxplot represents the distribution of sumscores across participants. The x-axis is empty, indicating no specific grouping, whilst the y-axis represents the sumscores. The data points are presented by boxes with varying heights and spreads, showing the variability in sumscores within each domain. For the Cognitive ability domain, it shows six potential outliers

Cattell’s scree-plot identified three factors with eigenvalues > 1.0 (14.4, 2.97, and 1.23), whilst Horn’s parallel analysis suggested a five-factor solution (Fig. 1). We, therefore, explored 5, 4, and 3-factor solutions accordingly. The five-factor solution lacked conceptual clarity and interpretability, failing to align with the theoretical framework to guide the constructs of interest. Moreover, the five-factor solution had limited practical implications and had too few items to form a fifth factor, hence being disproportionate; therefore, this solution was abandoned. The four-factor solution was explored by assessing factor loading, communalities, and conceptual coherence. This analysis gave a more interpretable and conceptually coherent factor structure, enhancing the differentiation and labelling of the underlying factors. However, challenges persisted regarding item placement and cross-loading. A final 3-factor solution was explored to address these concerns. The 3-factor models were iterated five times until no items were identified as single items (Table 3). The final 3-factor solution demonstrated favourable factor loading, communalities, and conceptual coherence, offering a more concise representation of the underlying constructs without compromising meaningful interpretation and practical applicability. In addition, it explained 59% of the total variance of the WORQ, whereas the five-factor solution explained 53% and the four-factor solution explained 57%, respectively (data not shown). In the final iteration, four items cross-loaded. Item 23 was placed in factor 3 because of its significant factor loading. Despite having borderline factor loading, three items were retained due to their strong alignment with a specific factor. Item 5 was placed in factor 1, whereas items 9 and 10 cross-loaded equally into factors 1 and 3. After careful consideration, they were assigned to factor 3 because of clinical reasoning and their alignment with the underlying ICF concept. Thus, the three-factor solution ended up comprising three distinct factors: psychological wellbeing (items: 4 (sad/depressed), 5 (worried/anxious), 6 (irritable), 7 (temper), and 8 (self-confidence)), physical functioning (items: 12 (balance), 13 (pain), 14 (endurance), 15 (muscle strength), 22 (daily activities), 27 (lifting < 5 kg), 28 (lifting > 5 kg), 30 (walking < 1 km), 31 (walking > 1 km), and 32 (running)), and cognitive ability (items: 9 (thinking clearly), 10 (problem-solving), 17 (acquiring skills), 18 (attention), 19 (reading), 20 (decision-making), 23 (stress-handling), 24 (reading body signals), 25 (conversation), and 26 (using communication devices)). The three factors exhibited correlations ranging from 0.42 to 0.54 with each other, and their Cronbach’s alpha values ranged from 0.88 to 0.93, and McDonald’s omega values ranged from 0.92 to 0.96 (Table 3). Fifteen items had limited associations with the three identified factors. They were kept as screening items to maintain a thorough biopsychosocial evaluation of functioning in accordance with the ICF framework. This approach guarantees a comprehensive understanding of an individual’s functioning, which aligns with the intended purpose of WORQ. No outliers were identified in the psychological wellbeing or physical functioning domain. Notably, in the cognitive ability domain, six individuals had scores defining them as potential outliers (Fig. 2). In the sensitivity analysis without these individuals, the EFA results remained consistent, affirming the stability of our findings.

**Table 3:** Exploratory Factor Analysis (EFA) Results for the Work Rehabilitation Questionnaire (WORQ): Unveiling Dimensionality in Assessing Psychological Wellbeing, Physical Functioning, and Cognitive Ability among patients with low back pain

Item	All items						Removing items: 5 single items Loading ≤ 0.3: items 11, 16 Communality < 0.25: items 38–40						Removing items: 8 single items Loading ≤ 0.3: items 11, 16 Communality < 0.25: items 38–40 Poor loading and communality: items 2, 29, 36					
	Factor 1	Factor 2	Factor 3	Communality	Uniqueness	Complexity	Factor 1	Factor 2	Factor 3	Communality	Uniqueness	Complexity	Factor 1	Factor 2	Factor 3	Communality	Uniqueness	Complexity
1	0.42	0.42		0.48	0.52	2.1	0.34	0.45		0.48	0.52	1.9	0.31	0.43		0.46	0.54	1.9
2		0.40		0.35	0.65	1.9		0.42		0.35	0.65	1.6						
3	0.50			0.49	0.51	1.4			0.39	0.49	0.51	2.3			0.42	0.49	0.51	2.0
4	0.65			0.54	0.46	1.1	0.49			0.54	0.46	1.7	0.49			0.56	0.44	1.7
5	0.58			0.54	0.46	1.2	0.42		0.34	0.54	0.46	2.1	0.42		0.34	0.55	0.45	2.1
6	0.86			0.65	0.35	1.1	0.81			0.72	0.28	1.0	0.85			0.76	0.24	1.0
7	0.78			0.52	0.48	1.0	0.73			0.59	0.41	1.0	0.76			0.62	0.38	1.0
8	0.74			0.59	0.41	1.0	0.55			0.59	0.41	1.5	0.52		0.30	0.58	0.42	1.7
9	0.74			0.70	0.30	1.1	0.48		0.46	0.70	0.30	2.0	0.44		0.50	0.69	0.31	2.0
10	0.68			0.66	0.34	1.2	0.43		0.47	0.66	0.34	2.0	0.40		0.50	0.65	0.35	1.9
11				0.14	0.86	1.6												
12		0.58		0.40	0.60	1.1		0.58		0.39	0.61	1.0		0.57		0.39	0.61	1.0
13		0.64		0.56	0.44	1.5		0.66		0.55	0.45	1.3		0.65		0.54	0.46	1.2
14		0.72		0.62	0.38	1.1		0.73		0.61	0.39	1.1		0.73		0.61	0.39	1.0
15		0.60		0.46	0.54	1.1		0.61		0.46	0.54	1.1		0.61		0.46	0.54	1.1
16				0.19	0.81	1.9												
17			0.56	0.52	0.48	1.4			0.76	0.55	0.45	1.0			0.79	0.56	0.44	1.0
18	0.50		0.37	0.63	0.37	1.9			0.65	0.65	0.35	1.2			0.68	0.66	0.34	1.1
19			0.55	0.52	0.48	1.3			0.75	0.55	0.45	1.0			0.78	0.57	0.43	1.0
20	0.42		0.48	0.61	0.39	2.0			0.72	0.63	0.37	1.1			0.76	0.64	0.36	1.0
21		0.31	0.32	0.53	0.47	3.0		0.31	0.46	0.53	0.47	1.9		0.31	0.46	0.53	0.47	1.9
22		0.62		0.56	0.44	1.2		0.63		0.57	0.43	1.1		0.62		0.57	0.43	1.2
23	0.64			0.66	0.34	1.3	0.36		0.57	0.67	0.33	1.7	0.32		0.60	0.68	0.32	1.5
24			0.54	0.58	0.42	1.6			0.70	0.56	0.44	1.0			0.70	0.55	0.45	1.0
25	0.31		0.52	0.56	0.44	1.6			0.74	0.58	0.42	1.0			0.74	0.58	0.42	1.0
26			0.72	0.52	0.48	1.0			0.80	0.51	0.49	1.2			0.78	0.48	0.52	1.2
27		0.80		0.58	0.42	1.1		0.79		0.59	0.41	1.1		0.79		0.59	0.41	1.1
28		0.81		0.60	0.40	1.0		0.82		0.60	0.40	1.0		0.82		0.60	0.40	1.0
29		0.30	0.35	0.32	0.68	2.0			0.35	0.29	0.71	1.9						
30		0.69		0.46	0.54	1.1		0.68		0.45	0.55	1.0		0.68		0.45	0.55	1.0
31		0.75		0.53	0.47	1.0		0.76		0.53	0.47	1.0		0.77		0.54	0.46	1.0
32		0.80		0.61	0.39	1.1		0.82		0.61	0.39	1.0		0.82		0.61	0.39	1.0
33		0.51		0.34	0.66	1.6		0.48		0.34	0.66	1.8		0.48		0.33	0.67	1.6
34		0.49		0.29	0.71	1.4		0.47		0.29	0.71	1.5		0.47		0.28	0.72	1.4
35		0.53		0.38	0.62	1.1		0.53		0.38	0.62	1.1		0.53		0.37	0.63	1.1
36	0.38			0.33	0.67	1.7				0.33	0.67	2.9						
37	0.46			0.45	0.55	1.6		0.40	0.40	0.44	0.56	2.0			0.42	0.43	0.57	1.8
38				0.19	0.81	1.6												
39			0.47	0.19	0.81	1.1												
40			0.52	0.24	0.76	1.0												
Crohnbach alpha	0.94	0.93	0.89				0.92	0.93	0.94				0.92	0.92	0.94			
McDonald’s omega	0.95	0.94	0.92				0.95	0.94	0.96				0.95	0.94	0.96			
Variance explained	0.19	0.18	0.11				0.12	0.21	0.19				0.12	0.21	0.21			
Cumulative variance	0.19	0.37	0.48				0.12	0.33	0.52				0.12	0.33	0.54			

Item	Removing items: 10 single items Loading ≤ 0.3: items 11, 16 Communality < 0.25: items 38–40 Poor loading (< 0.5) and communality (< 0.40): items 2, 29, 33, 34, 36						Removing items: 15 single items Loading ≤ 0.3: items 11, 16 Communality < 0.25: items 38–40 Poor loading (< 0.5) and communality (< 0.40): items 2, 29, 33, 34, 36 Removing poor loadings (< 0.5) except item 5, 9 and 10: items 1, 3, 21, 35, 37					
	Factor 1	Factor 2	Factor 3	Communality	Uniqueness	Complexity	Psychological well-being	Physical functioning	Cognitive ability	Communality	Uniqueness	Complexity
1		0.44		0.45	0.55	1.9						
2												
3			0.43	0.49	0.51	1.9						
4	0.50			0.56	0.44	1.7	**0.52**			0.56	0.44	1.6
5	0.45		0.33	0.55	0.45	2.0	**0.47**		*0.31*	0.56	0.44	1.9
6	0.87			0.77	0.23	1.0	**0.88**			0.77	0.23	1.0
7	0.77			0.62	0.38	1.0	**0.80**			0.64	0.36	1.0
8	0.52		0.30	0.58	0.42	1.7	**0.53**			0.58	0.42	1.6
9	0.42		0.51	0.69	0.31	1.9	*0.43*		**0.49**	0.67	0.33	2.0
10	0.41		0.49	0.65	0.35	1.9	*0.43*		**0.46**	0.64	0.36	2.0
11												
12		0.56		0.38	0.62	1.0		**0.55**		0.37	0.63	1.1
13		0.66		0.54	0.46	1.2		**0.64**		0.52	0.48	1.1
14		0.75		0.64	0.36	1.0		**0.75**		0.64	0.36	1.0
15		0.63		0.48	0.52	1.1		**0.63**		0.48	0.52	1.1
16												
17			0.82	0.58	0.42	1.0			**0.82**	0.60	0.40	1.0
18			0.71	0.67	0.33	1.1			**0.69**	0.67	0.33	1.1
19			0.81	0.59	0.41	1.1			**0.80**	0.59	0.41	1.1
20			0.77	0.65	0.35	1.0			**0.75**	0.64	0.36	1.0
21			0.44	0.52	0.48	2.0						
22		0.60		0.56	0.44	1.2		**0.58**		0.52	0.48	1.2
23	0.30		0.61	0.68	0.32	1.5	*0.33*		**0.59**	0.68	0.32	1.6
24			0.71	0.56	0.44	1.0			**0.69**	0.56	0.44	1.0
25			0.72	0.57	0.43	1.0			**0.70**	0.56	0.44	1.0
26			0.75	0.44	0.56	1.2			**0.75**	0.45	0.55	1.1
27		0.79		0.59	0.41	1.1		**0.78**		0.59	0.41	1.1
28		0.85		0.64	0.36	1.0		**0.85**		0.65	0.35	1.0
29												
30		0.66		0.44	0.56	1.0		**0.65**		0.43	0.57	1.0
31		0.77		0.55	0.45	1.0		**0.77**		0.56	0.44	1.0
32		0.84		0.63	0.37	1.0		**0.83**		0.64	0.36	1.0
33												
34												
35		0.48		0.35	0.65	1.3						
36												
37			0.40	0.43	0.57	1.9						
38												
39												
40												
Crohnbach alpha	0.92	0.92	0.94				0.88	0.92	0.93			
McDonald’s omega	0.95	0.95	0.96				0.93	0.94	0.96			
Variance explained	0.13	0.21	0.22				0.14	0.22	0.23			
Cumulative variance	0.13	0.34	0.55				0.14	0.36	0.59			

Italic numbers indicate cross-loadings

Bold numbers indicate items included in the domain

## Discussion

This study evaluated the item characteristics, factor structure, and internal consistency of the WORQ in individuals with LBP in an outpatient hospital care setting. Three key factors emerged: psychological wellbeing, physical functioning, and cognitive ability, which together provide a comprehensive framework for understanding the potential impact of LBP on workability, explaining more than half of the variance. Moreover, the factors’ item characteristics and strong internal consistency support the reliability.

The psychological wellbeing factor captures psychological aspects influencing work engagement and performance. Patients with LBP often experience elevated levels of psychosocial distress, including anxiety, depression, and somatisation, compared to those without [33]. These adverse impacts underscore the importance of integrating psychological factors into managing LBP. By comprehensively assessing psychological wellbeing, healthcare providers can gain insights into patients’ emotional states, stress management abilities, and motivational levels. This information can facilitate tailoring personalised interventions and support strategies, acknowledging and addressing the intricate impact psychological components have on workability.

Despite the overt impairments in physical function and the consequential threat to independence posed by persistent LBP, there is a significant discrepancy between individuals’ perceived and objectively measured physical functioning [34]. Notably, patients with LBP may overpredict functional declines due to pain and fear avoidance, contributing to the adverse impact on physical functioning in various contexts [35]. However, it is important to acknowledge that these perceived limitations are intrinsic to the individual and are not influenced by the assessment instrument or their specific condition. They exist with greater or lesser intensity, independent of external factors. It emphasises the complex relationship between LBP-perceived functional limitations and objective physical performance. Given the complexity, a comprehensive approach is important when assessing physical functioning. Including work-related questions in the evaluation helps healthcare providers better understand the decline in functioning caused by the condition, allowing for tailored interventions and support strategies to address individual needs.

The cognitive ability factor measures cognitive functions, including concentration and problem-solving skills. These cognitive impairments can be influenced by pain perception and contribute to disability [36]. Recognising and understanding these cognitive aspects is important for developing effective pain management strategies and addressing psychological wellbeing. By incorporating cognitive assessments, such as those offered by the WORQ, healthcare providers can identify individuals who may benefit from cognitive rehabilitation strategies to optimise their work performance. This personalised approach may lead to tailored treatment plans, improving functional outcomes and enhancing wellbeing.

The founding WORQ publication classified items into four factors: emotion (6 items), cognition (10 items), dexterity (10 items), and mobility (4 items), along with 10 single items. However, seven factors were identified in a Dutch version that was translated and culturally adapted for patients with fibromyalgia and hand and wrist injuries [13]. Our study supports a multidimensional model but also suggests that a model with three factors can effectively assess limitations in work function. The three-factor structure offers a more focused and conceptually coherent representation of work functioning in individuals with persistent LBP. The original four-factor structure included a “dexterity” domain, which may not be relevant for individuals with LBP. However, retaining a “cognitive ability” domain is a distinctive feature of our study that addresses an interesting aspect often overlooked in LBP-specific questionnaires. Therefore, we recommend that the WORQ with its generic origin are validated towards the specific population investigated. The variability in the findings of different factor structures may be attributed to different patient populations and cultural perspectives. Moreover, variations in data analytic approaches and the influence of sample sizes may have affected the results. Nonetheless, regardless of the specific factors or subscales, the WORQ can comprehensively assess work functioning from a biopsychosocial aspect and the alignment with the ICF framework may facilitate effective communication and collaboration amongst professionals, promoting knowledge exchange in work rehabilitation and reducing work disability.

Whilst 15 items did not meet the criteria for inclusion in the identified factors, retaining them holds potential value in assessing work limitations beyond these factors. This retention aligns to conduct a comprehensive assessment that acknowledges the multidimensional nature of health and functioning, ultimately enabling a more individualised rehabilitation. However, it is important to acknowledge that other suitable instruments may be used together with WORQ for assessing specific conditions or outcomes than these 15 items alone. In such instances, it is recommended to employ validated instruments specifically designed for those purposes. By utilising a combination of appropriate instruments, it becomes possible to attain a more accurate and comprehensive understanding of individuals’ work functioning across multiple domains. Identifying factors and single items may raise concerns about an aggregated score of all items’ clinical applicability, as it assumes equal weight for all items and disregards variations in specific factors or individual items [37]. Our results show that certain items primarily capture psychological wellbeing, whilst others capture physical functioning or cognitive ability. By assigning equal weight to all items, an aggregated score fails to differentiate between these distinct dimensions and may overlook important variations in specific areas of workability. Consequently, prioritising domain scores to guide targeted interventions rather than relying solely on an aggregated overall score may be beneficial to capture the worker’s workability or capacity fully.

## Strengths and Limitations

Incorporating participants from a hospital care setting improves the sample’s representativeness as it reflects individuals actively seeking treatment for LBP in genuine hospital care environments. However, it is important to note that the direct extrapolation of our findings to primary care settings may require further investigation. The handling of the six outliers identified in the cognitive ability domain holds merit. These individuals were carefully considered as they have the potential to influence factor loadings and, consequently, the accurate representation of domains. Their presence may also influence correlations, potentially affecting the overall factor structure [19]. To address this, we conducted a sensitivity analysis by performing the EFA with and without these outliers, ensuring the stability of our findings. These findings collectively bolster the credibility and validity of our study’s outcomes. Interpreting the factor structure obtained from EFA can be challenging, as it involves a partly subjective determination of the appropriate placement of certain items and careful identification of potential cross-loadings. We have transparently documented and reported the factor analysis process and explained the process behind retaining items with borderline factor loadings and communalities to ensure the replicability of our study. Justification for retaining such items has been provided based on their theoretical congruence and alignment with the ICF. It is important to acknowledge that alternative interpretations of the factor structure may exist, and caution should be exercised when interpreting the results in different populations and care settings. Our choice to employ EFA instead of Confirmatory Factor Analysis (CFA) was driven by the exploratory nature of our research question, enabling us to uncover specific nuances. In moving forward, opportunities for refinement exist. Future research could assess item discrimination and difficulty parameters, employing Item Response Theory for a more detailed item-level assessment. Additional validation through CFA, responsiveness metrics, and group-specific analyses could augment the psychometric attributes of the WORQ. Investigations into convergent and discriminant validity and longitudinal and demographic variations are also pivotal aspects to contemplate for a comprehensive assessment.

A study limitation is the potential lack of generalizability. Replicating the study in diverse settings would be necessary to establish the external validity of the identified dimensions. Moreover, the decision to include 400 respondents was driven by practical considerations using a rule-of-thumb principle without formal sample size estimation. Although this approach is in line with conventional practices, it is important to recognise that sample size estimation involves multiple considerations, and the adequacy of the sample size remains uncertain. Another limitation arises from the reliance on self-reported measures for determining inclusion and exclusion criteria, introducing the potential for participant selection bias. However, including a non-responder analysis provides confidence that any selection bias is likely minimal in this study.

### Clinical Implications

The identification of three factors in the WORQ underscores the complexity of workability in individuals with LBP, aligning with the biopsychosocial model. This insight equips healthcare professionals to tailor rehabilitative interventions. As depicted in the boxplot (Fig. 2), our population was mostly affected in their physical functioning and psychological wellbeing. How this information is best translated into clinical practice must rely on an individual clinical evaluation. Nonetheless, we find that the WORQ holds promise for structured communication in rehabilitation, aiming to optimise work performance for individuals with persistent LBP. Still, validation across diverse populations and settings, alongside exploring variables like work absenteeism and presenteeism (at-work productivity loss), is needed.

## Conclusion

WORQ is a valid instrument for assessing work limitations in individuals with persistent LBP. The EFA identified three key factors: psychological wellbeing, physical functioning, and cognitive ability. These factors align with the common challenges that patients with LBP face and support the use of the ICF framework. Further research should assess its responsiveness to changes from interventions that target workability. Advancing this knowledge has the potential to promote work rehabilitation and improve the quality of life for patients with persistent LBP.

## Data Availability

The datasets generated during and/or analysed during the current study are available from the corresponding author on reasonable request.
